# Should Kidney Transplantation be Offered to Patients with Body Mass Index >40?: Commentary

**DOI:** 10.34067/KID.0000000954

**Published:** 2025-08-08

**Authors:** Phillipe Abreu, Jesse D. Schold

**Affiliations:** 1 Department of Surgery, University of Colorado–Anschutz Medical Campus, Aurora, Colorado; 2 Department of Epidemiology, School of Public Health, University of Colorado–Anschutz Medical Campus, Aurora, Colorado

**Keywords:** kidney transplantation, outcomes, risk factors, transplantation

Obesity continues to rise as a global health concern and significantly affects access to kidney transplantation. Exclusion of high-body mass index (BMI) candidates for transplantation emerged from observational studies in the 1990s and early 2000s, which associated severe obesity with increased perioperative risks, wound dehiscence, surgical site infections, incisional hernias, and graft rejection.^1^ Additional immunosuppression required to prevent graft rejection increases infection risks, thereby often necessitating reductions in immunosuppression, subsequently elevating the risk of graft loss.^1^ A recent national survey highlighted that approximately 73% of US transplant programs uphold BMI thresholds of 40 kg/m^2^ at referral or waitlisting, despite a paucity of evidence to support practice.^1^

**Figure 1:**
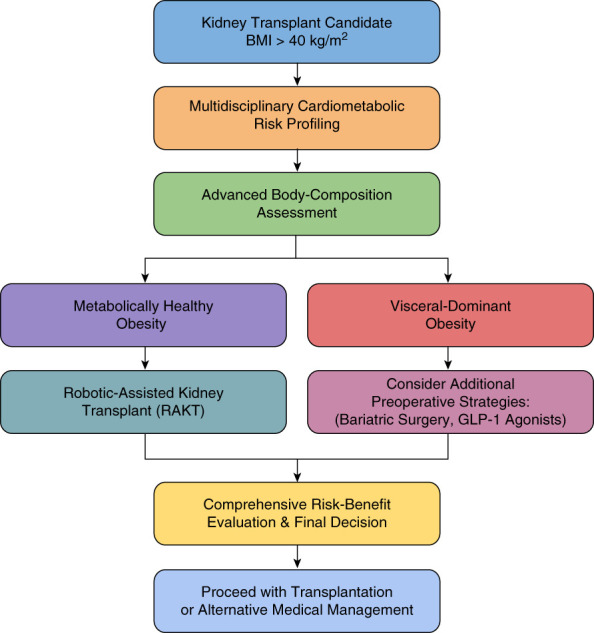
**Decision algorithm for evaluating and managing kidney transplant candidates with a BMI>40 kg/m**^**2**^**.** The flow chart integrates multidisciplinary cardiometabolic profiling, comprehensive body composition analysis, surgical feasibility (favoring RAKT), and strategic consideration of bariatric or metabolic interventions when appropriate. BMI, body mass index; RAKT, robotic-assisted kidney transplantation; SG, sleeve gastrectomy.

The CON argument for transplantation of high-BMI candidates, presented in this issue by Rita McGill, emphasizes increased risks associated with kidney transplantation in severely obese patients, including surgical complications, delayed graft function, graft failure, and mortality.^2^ McGill underscores that risks persist despite advances in transplantation methods and are particularly pronounced for patients with class-3 obesity (BMI >40 kg/m^2^). Additionally, McGill notes ethical considerations related to fair and optimal allocation of scarce donor organs, suggesting that transplantation in this high-risk group compromises both individual patient outcomes and broader organ distribution fairness. Thus, the CON perspective advocates caution and prioritization of resources toward patients likely to derive maximum benefit with minimal risk.

Conversely, the PRO argument, presented by Amanda Vinson in this issue, emphasizes the substantial survival advantage kidney transplantation provides to patients with BMI >40 kg/m^2^ compared with dialysis.^3^ Vinson highlights significant limitations of BMI for risk assessment, noting insufficient differentiation between muscle, bone, and fat mass nor accounting for body fat distribution, crucial for accurately assessing metabolic risk. Moreover, Vinson stresses that BMI thresholds exacerbate health inequities, arguing for individualized assessments rather than uniform BMI-based eligibility cutoffs. Thus, from the PRO perspective, restricting transplantation based on BMI thresholds is not only clinically counterproductive but also ethically questionable.

Current evidence differentiates between modifiable and nonmodifiable risk factors.^2^ For instance, obese patients undergoing dialysis frequently demonstrate paradoxically better survival rates compared with their leaner counterparts because of enhanced metabolic reserves, an observation termed reverse epidemiology.^4^ Additionally, the association of BMI with post-kidney transplant outcomes is significantly modified by factors including patient age, race/ethnicity, sex, and primary diagnosis.^5^ Although subcutaneous fat contributes to higher incidence of wound infections because of poor vascularization, surgical variables such as incision size, meticulous tissue handling, and ischemia time remain modifiable and controllable factors.

Importantly, significant advances in minimally invasive surgical techniques, particularly robotic-assisted kidney transplantation (RAKT), have altered this risk-benefit evaluation. Initially introduced for donor nephrectomies, robotic techniques have expanded to recipient procedures, significantly reducing perioperative morbidity. Tzvetanov *et al.* demonstrated that among 239 RAKT recipients with median BMI=41.4 kg/m^2^, surgical site infection rates were remarkably low (0.4%), with a 3-year patient survival rate of 95%.^6^ Similarly, Tinney *et al.* confirmed that RAKT significantly decreased major postoperative complications compared with traditional open kidney transplantation, reducing Clavien–Dindo grade ≥3 complications nearly five-fold.^7^ Spaggiari *et al.* extended findings by successfully implementing RAKT with deceased donor kidneys, including a 5-year death-censored graft survival of 86%, surpassing matched cohorts undergoing traditional transplantation techniques, with particular benefit for obese patients.^8^

Despite surgical advances, alternative risk mitigation strategies, such as bariatric surgery and medical therapies, have limitations. Although sleeve gastrectomy effectively induces substantial weight loss, adoption among patients with ESKD is limited, with <5% completing the bariatric pathway, predominantly because of operative and perioperative concerns.^6^ GLP-1 agonists require extended durations for substantial weight loss and are often poorly tolerated in dialysis-dependent patients, while being associated with insurance barriers and cost limitations. Traditional methods, such as intensive nutritional counseling and exercise interventions, are severely constrained by the physical toll and scheduling demands of dialysis. Hence, mandated weight loss often proves impractical and may unintentionally prolong dialysis duration, increasing cardiovascular morbidity and mortality with unclear benefits.^9^

BMI fails to differentiate muscle from fat mass and neglects critical elements such as visceral adiposity distribution and metabolic health markers. Ayuzo Del Valle *et al.* recently proposed a nuanced framework integrating comprehensive anthropometric and metabolic evaluations. This obesity phenotype assessment delineates individuals with metabolically healthy obesity who, despite high BMI, demonstrate favorable insulin sensitivity and minimal hepatic steatosis, thus potentially benefiting from transplantation.^10^ Conversely, individuals characterized by visceral obesity carry heightened cardiovascular risks and may require additional surgical considerations. Adopting such phenotype-driven assessments enables more accurate and equitable patient selection.

A historical concern in high-BMI transplantation relates to organ-to-recipient size mismatches. Recent data involving 112 living-donor recipient pairs highlighted that renal parenchymal volume adjusted for patient weight better predicts long-term graft function than absolute kidney size.^11^ This finding challenges traditional assumptions that kidneys are too small for obese recipients and underscores nuanced considerations based on lean body mass. Economic and equity considerations support arguments against strict BMI-based exclusion. Dialysis imposes significant economic burdens, with annual costs per patient surpassing $90,000 USD.^12^ Thus, delaying transplantation to pursue weight loss strategies often negates potential savings from reduced surgical complications. Moreover, strict BMI thresholds disproportionately affect ethnic minorities and socioeconomically disadvantaged groups, exacerbating health care inequities. Obese patients often spend disproportionately prolonged periods on dialysis in transplant evaluations primarily because of BMI cutoffs. When these patients are eventually listed for transplantation, they generally receive priority for high-quality deceased donor kidney organ offers, underscoring critical need for improved access strategies such as robotic transplantation to optimize outcomes.^8^

Cumulatively, evidence suggests that programs should reconsider strict BMI cutoffs, replacing them with comprehensive multidisciplinary assessments integrating metabolic health, body composition metrics, and surgical feasibility. RAKT can be expanded, facilitating timely utilization of high-quality deceased donor organs. Bariatric procedures can be selectively integrated based on patient profiles, without serving as prerequisites for transplant candidacy. Figure 1 exemplifies a comprehensive decision-making algorithm for this population. Prospective data collection through national consortia could refine existing risk calculators, enhancing individualized patient assessments. Additionally, engagement with health care payers highlighting cost-effectiveness of timely transplantation over prolonged dialysis may be effective. Future research is needed prioritizing randomized controlled trials comparing RAKT to traditional methods, validating comprehensive body composition-based selection algorithms and evaluating long-term outcomes after robotic transplantation. Incorporating artificial intelligence and imaging techniques could further optimize donor–recipient size matching. By adopting such progressive, comprehensive approaches, the transplant community can ensure fairer, more effective patient care and address historical disparities inherent in strict BMI-based exclusion criteria.
